# *In Vitro* Antiplasmodial Activity of Sesquiterpene Lactones from *Ambrosia tenuifolia*

**DOI:** 10.1155/2011/352938

**Published:** 2011-05-25

**Authors:** V. Sülsen, D. Gutierrez Yappu, L. Laurella, C. Anesini, A. Gimenez Turba, V. Martino, L. Muschietti

**Affiliations:** ^1^Cátedra de Farmacognosia, IQUIMEFA (UBA-CONICET), Facultad de Farmacia y Bioquímica, UBA, Junín 956, Buenos Aires 1113, Argentina; ^2^Instituto de Investigaciones Fármaco Bioquímicas, Facultad de Ciencias Farmacéuticas y Bioquímicas, Universidad Mayor de San Andrés, Avenida B. Saavedra 2224, La Paz 3239, Bolivia

## Abstract

The *in vitro* antiplasmodial activity of *Ambrosia tenuifolia* organic extract and its isolated sesquiterpene lactones, psilostachyin and peruvin, has been evaluated against *Plasmodium falciparum* F32 and W2 strains. The cytotoxicity of both compounds was determined on lymphoid cells, and their corresponding selectivity indexes (SIs) were calculated. Peruvin was the most active compound on F32 strain of *P. falciparum* with a 50% inhibitory concentration value (IC_50_) of 0.3 **μ**g/mL (1.1 **μ**M) whereas psilostachyin showed activity on both strains (IC_50_ = 0.6 (2.1 **μ**M) and 1.8 **μ**g/mL (6.4 **μ**M)). Fifty percent cytotoxic concentration (CC_50_) values (48 h) were 6.8 **μ**g/mL (24.3 **μ**M) and 10.0 **μ**g/mL (37.9 **μ**M) for psilostachyin and peruvin, respectively.

## 1. Introduction

Malaria is a major parasitic infection in tropical and subtropical regions of the world, particularly devastating in sub-Saharan Africa where 90% of the cases and deaths occur [1]. According to the World Health Organization (WHO), over a million people die from malaria every year, and roughly a 40% of the population is at risk of becoming infected. In America, this infection spreads from Northern to Southern America, being Brazil and the Andes Region, the areas where the greatest number of cases is recorded [2]. 

Medicinal plants have provided valuable antimalarial drugs such as quinine and artemisinin. The discovery of these drugs, which are currently being used, has prompted the evaluation of other medicinal plants in the search of new antimalarial agents that are not only active against drug-sensitive but also against drug-resistant and multidrug-resistant strains of *Plasmodium falciparum*. Although artemisinin, a sesquiterpene lactone (STL), is the most powerful drug against chloroquine-resistant malaria, resistance to this drug might soon appear. Therefore, there is an urgent need for new therapeutic agents [3].

In previous studies we have demonstrated that the CH_2_Cl_2_ : MeOH (1 : 1) of *Ambrosia tenuifolia* has a significant trypanocidal activity against *Trypanosoma cruzi *epimastigotes [4]. Besides, its essential oil showed antiplasmodial activity against chloroquine-sensitive and chloroquine-resistant strains of *P. falciparum* [5].* A. tenuifolia* Sprengel (Asteraceae) is an Argentine medicinal species known as “ajenjo del campo,” “altamisa,” or “artemisia” which is traditionally used for the treatment of intermittent fevers [6, 7]. 

**Figure 1:**
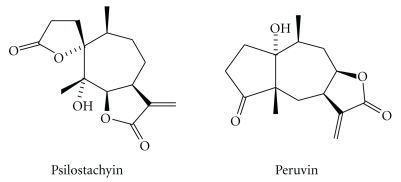
Chemical structure of sesquiterpene lactones isolated from *Ambrosia tenuifolia*.

A bioguided assay fractionation of the CH_2_Cl_2_ : MeOH (1 : 1) extract of *A. tenuifolia* led to the isolation of two STLs, belonging to the pseudoguaianolide type, identified as psilostachyin and peruvin (Figure 1). Both compounds were active against *T. cruzi* and showed antileishmanial activity against *Leishmania mexicana* promastigotes [8]. The effect of psilostachyin on the growth of *T. cruzi *epimastigotes with the addition of glutathione and at the ultrastructural level of the parasite has been reported [9]. 

These findings prompted us to assess the activity of *A. tenuifolia* organic extract and its isolated compounds, psilostachyin and peruvin, on both chloroquine-sensitive (F32) and chloroquine-resistant strains (W2) of *P. falciparum.* Cytoxicity of such compounds on lymphoid cells has been also tested.

## 2. Materials and Methods

### 2.1. Plant Material

The aerial parts of *A. tenuifolia* were collected in Punta Lara, Province of Buenos Aires, Argentina, in April 2007. The plant material was identified by Dr. G. Giberti, and a voucher specimen (BAF 660) is deposited at the Herbarium of the Museo de Farmacobotánica, Facultad de Farmacia y Bioquímica, Universidad de Buenos Aires.

### 2.2. Extraction of Plant Material

Dried aerial parts (1000 g) of *A. tenuifolia* were extracted with CH_2_Cl_2_ : MeOH (1 : 1) (10 L) at room temperature for 24 h and vacuum filtered. The process was repeated twice, and the filtrates were combined and dried under vacuum.

### 2.3. Isolation of Psilostachyin and Peruvin

Psilostachyin and peruvin were isolated from the organic extract of *A. tenuifolia* as previously described [8]. The purity of psilostachyin and peruvin was confirmed by High Performance Liquid Chromatography (HPLC) : HPLC-DAD (Waters), gradient H_2_O : MeOH 0–70% for 20 min, 70–100% for 10 min; C_18_ column (LiChrospher 5 microns, 125 × 4 mm, Merck) flow 1.0 mL/min.

### 2.4. Parasite Strains and Culture Media

*Plasmodium falciparum* F32-Tanzania strain (chloroquine sensitive, kindly provided by Dr. Fandeur T, Pasteur Institute, Kayenne) and *P. falciparum* W2 strain (chloroquine resistant, kindly provided by Dr Eduardo Ortega Barria, INDICASAT Institute, Panamá) were cultured in RPMI 1640 medium supplemented with 10% human serum and a hematocrit of 4% (Blood group O, RH+) at 37°C in an anaerobic atmosphere [10].

### 2.5. Animals

Inbred female BALB/c mice (22 ± 2 g) were purchased from the Instituto Nacional de Tecnología Agropecuaria (INTA). Animals were kept according to the Guide for the Care and Use of Laboratory Animals, US National Research Council [11].

### 2.6. In Vitro Antiplasmodial Activity Assay

Parasite growth was synchronized at 1% parasitemia and 2% hematocrite and distributed in a volume of 100 *μ*L in plates of 96 wells by duplicate. Antiplasmodial activity was evaluated in concentrations between 100 and 0.01 *μ*g/mL for both the organic extract and isolated compounds. 100 *μ*L of each dilution (in DMSO, at no more than 0.1% final concentration) was added to each well. Parasites were then incubated at 37°C for 48 h. After incubation, smears were prepared, fixed with methanol, and stained with Giemsa. The antiplasmodial activity was determined by microscopy counting of noninfected red cells and infected red cells. The 50% inhibitory concentrations (IC_50_) of the extract and the compounds were calculated graphically using CRICKET GRAPH 1.3 software. Chloroquine (10–1000 nM) (Sigma-Aldrich) was used as a positive control, and DMSO was employed as negative control. All assays were carried out in triplicate. 

### 2.7. Cytotoxicity Assay

Lymphoid cell suspensions were obtained from the lymph nodes of BALB/c mice as previously reported [8]. Briefly, T-cell enriched populations were obtained by passage of the cell suspension through a nylon wool column. T-cell presence was higher than 97% as checked by indirect immunofluorescence after lysis with anti-Thy plus complement. Cells were cultured at a concentration of 2 × 10^6^ cells/mL in RPMI 1640 medium (GIBCO) supplemented with 10% fetal calf serum, 2 mM glutamine, 100 U/mL penicillin, and 100 *μ*g/mL streptomycin. Cells were adjusted to a final volume of 0.2 mL per well in a 96-well microtiter plate and cultured at 37°C in a 5% CO_2_ atmosphere for 3, 24, and 48 h. Cell viability was determined by the trypan blue exclusion method in the absence and presence of increasing concentrations of the compounds (0.1, 1, 10, and 50 *μ*g/mL) and was expressed as % of basal values, calculated as [number of viable cells/number of total cells] × 100. Then, data of viability were transformed in cytotoxicity values (100-% of viability) to calculate the 50% cytotoxic concentration (CC_50_) which was finally calculated using CRICKET GRAPH 1.3 software. The tests were performed in triplicate. The selectivity index (SI) was used to compare the toxicity for mammalian cells and the activity against the parasites and calculated as the CC_50_ on murine T lymphocytes (48 h) divided by the 50% inhibitory concentration (IC_50_) of the compound for both strains of *P. falciparum.*

## 3. Results and Discussion

The organic extract of* Ambrosia tenuifolia* was assayed *in vitro *on *P. falciparum* F32 strain. This extract showed promising antiplasmodial activity with a growth inhibition of 77.1 ± 1.22% (10 *μ*g*/*mL), with an IC_50_ value of 2 *μ*g/mL (Table 1). Psilostachyin and peruvin, two STLs isolated from this extract, were tested for their ability to inhibit the same *P. falciparum* strain and a chloroquine-resistant strain (W2). These STLs were active on both parasite strains but showed stronger activity on the F32 strain. Peruvin was the most active compound on this strain with an IC_50_ value of 0.3 *μ*g*/*mL (1.1 *μ*M) whereas psilostachyin showed activity on both F32 and W2 strains with IC_50_ values of 0.6 (2.1 *μ*M) and 1.8 *μ*g*/*mL (6.4 *μ*M), respectively (Table 1).

**Figure 2:**
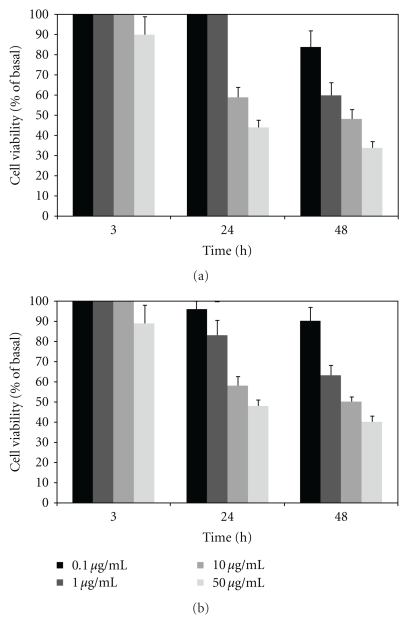
Effects of psilostachyin (a) and peruvin (b) on T-lymphocyte viability. Cultures were done in 96-well plates with 2 × 10^6^ lymphocytes/mL during 3, 24 or 48 h in the presence of different concentrations (0.1 to 50 *μ*g/mL) of the compounds. Cell viability was determined by the trypan blue exclusion method and is expressed as viability. Bars represent the means ± SEM.

The isolated compounds were evaluated for their cytotoxicity on T-cells at 3, 24 and 48 h (Figure 2). The CC_50_ values (48 h) for psilostachyin and peruvin and the SIs were calculated and are shown in Table 1.

This is the first report on the antiplasmodial activity of psilostachyin and peruvin. The IC_50_ values for these compounds were in the *μ*M range as it has been reported for other STLs [12] and particularly for pseudoguaianolides such as helenalin and its ester derivatives [13]. 

Antiplasmodial activity of this kind of compounds has been usually associated with high cytotoxicity [14, 15]. However, certain STLs, mainly from the pseudoguaianolide type, are considerably more toxic to the parasites than to mammalian cells [13]. These findings suggest that other structural features are governing the antiplasmodial activity of this kind of compounds besides the effect related to the *α*-methylene-*γ*-lactone group (Michael addition).

Since the discovery of artemisinin against chloroquine-resistant malaria, attention has been paid to other STLs as a potential source of antimalarial drugs. The results shown herein for psilostachyin and peruvin, on *P. falciparum*, make these molecules interesting scaffolds to generate leads with enhanced antiplasmodial activity and reduced cytotoxicity. Further investigation will include the *in vivo* studies and the molecular mechanism of action of these compounds.

## Figures and Tables

**Table 1:** *In vitro* antiplasmodial activity, cytotoxicity and selectivity indexes of *Ambrosia tenuifola* extract, psilostachyin and peruvin.

Extract/Compounds	IC_50_ (*μ*M)		CC_50_ (*μ*M)	Selectivity Index	
	*P*. *falciparum *	*P*. *falciparum *	48 h	*P*. *falciparum *	*P*. *falciparum *
	F32 strain	W2 strain		F32 strain	W2 strain
CH_2_Cl_2_ : MeOH (1 : 1)	2.0 ± 0.09*	n.t.	n.t.	n.c.	n.c.
Psilostachyin	2.1 ± 0.15	6.4 ± 0.48	24.3 ± 0.21	11.6 ± 0.95	3.8 ± 0.25
Peruvin	1.1 ± 0.09	18.9 ± 0.95	37.9 ± 0.34	34.4 ± 2.40	2.0 ± 0.15
Chloroquine	0.03 ± 0.002	2.8 ± 0.19	n.t.	n.c.	n.c.

n.t.: not tested; n.c.: not calculated.

*Expressed as *μ*g/ML.
